# Tracking aspects of healthcare activity during the first nine months of COVID-19 in Ireland: a secondary analysis of publicly available data

**DOI:** 10.12688/hrbopenres.13372.1

**Published:** 2021-09-02

**Authors:** Domhnall McGlacken-Byrne, Sarah Parker, Sara Burke

**Affiliations:** 1 Royal College of Physicians in Ireland, Kildare Street, Dublin 2, Ireland; 2 Centre for Health Policy and Management, Trinity College Dublin, Dublin, Dublin 2, Ireland

**Keywords:** Health reform, health system, health policy, Sláintecare, COVID-19, implementation, Ireland.

## Abstract

**Background**: Sláintecare aims to introduce universal healthcare in Ireland. The COVID-19 pandemic poses both challenges and opportunities to this process. This study explored the impact of COVID-19 on aspects of Irish healthcare during the first nine months of the pandemic and considers the implications for Sláintecare implementation.

**Methods**: Secondary analysis was undertaken on publicly available data on three key domains of the Irish healthcare system: primary care, community-based allied healthcare, and hospitals. Descriptive statistics were computed using Microsoft Excel 2016.

**Results**: Up to March 2021, 3.76 million COVID-19 tests were performed by Ireland’s public healthcare system, 2.48 million (66.0%) of which were referred from the community. General practitioners delivered 2.31 million telephone triages of COVID-19 symptoms, peaking in December 2020 when 416,607 consultations occurred. Patient numbers across eight allied healthcare specialties fell by 35.1% versus previous years, with the greatest reductions seen in speech and language therapy (49.0%) and audiology (46.1%). Hospital waiting lists increased from 729,937 to 869,676 (or by 19.1%) from January 2019 to January 2021. In January 2021, 629,919 patients awaited a first outpatient clinic appointment, with 170,983 (27.1%) waiting longer than 18 months. The largest outpatient lists were observed in orthopaedic surgery (n=77,257); ear, nose and throat surgery (n=68,073); and ophthalmology (n=47,075). The proportion of patients waiting more than 12 months for a day-case gastrointestinal endoscopy rose from 6.0% in January 2020 to 19.0% in January 2021.

**Conclusions**: Healthcare activity has been significantly disrupted by COVID-19, leading to increased wait times and greater barriers to healthcare access during the pandemic. Yet, Ireland’s health system responses also revealed strong willingness and ability to adapt and to implement novel solutions for healthcare delivery, rapidly and at scale. This has demonstrated what is achievable under Sláintecare and provides a unique opportunity to ‘build back better’ towards sustainable recovery.

## Introduction

The COVID-19 pandemic has had a profound negative impact on societies worldwide ( World Health Organisation, 2020a). Ireland is no exception to this, experiencing more than 250,000 cases of COVID-19 as of May 2021, over 4,900 deaths, and widespread disruption of non-COVID healthcare ( Government of Ireland, 2020a; Rosenbaum, 2020). The pandemic occurs within a period of strategic change within Irish healthcare ( Burke *et al.*, 2018; Department of Health/Sláintecare Implementation Office, 2018). Historically, Ireland’s health system has been undermined by flaws relating to capacity, equity, long wait times for scheduled hospital care, and fragmentation of services ( Burke *et al.*, 2016; Department of Health/Sláintecare Implementation Office, 2019; Organisation for Economic Co-Operation and Development, 2019). The Sláintecare reform process aims to transform Irish healthcare over a ten-year period, to a universal system of care that is equitable, timely and transitioned, where possible, to the community setting (‘right care, right place, right time’) ( Department of Health/Sláintecare Implementation Office, 2019; Houses of the Oireachtas, 2017).

The adverse effects of widespread cancellation of healthcare are self-evident. However, it has also been observed that certain strategic priorities of the national pandemic response may align with those of Sláintecare, particularly those relating to the transition of care out of hospitals; technological innovation; and removal of barriers to healthcare access ( Burke *et al.*, 2020; Health Service Executive, 2020a; Health Service Executive, 2021a; Health Service Executive, 2021b; National Public Health Emergency Team, 2020a). For example, while Ireland is currently the only country in Western Europe without universal access to primary care ( Organisation for Economic Co-Operation and Development, 2019), all COVID-related healthcare has remained free at the point of use during the pandemic, though a significant proportion of the population may be unaware of this ( Brick *et al.*, 2020).

The rapid adoption of agile responses, such as telemedicine, also provided universal access to remote general practitioner (GP) care for those presenting with COVID-19 symptoms ( Burke *et al.*, 2020). Moreover, insofar as the importance of allied health professionals (AHPs) to in-hospital care of COVID-19 patients has been recognised ( Coto *et al.*, 2020; Thomas *et al.*, 2020), significant numbers of AHPs were redeployed from their primary work to other activities, such as contact-tracing and testing ( Raidió Teilifís Éireann, 2020a; Raidió Teilifís Éireann, 2020b).

However, the impact of the pandemic has also prompted several changes in the healthcare system that may inhibit or pose challenges for the roll-out of Sláintecare in the longer-term. First, each ‘wave’ of the pandemic has resulted in extensive curtailment of elective activity in hospitals ( Government of Ireland, 2020b; National Public Health Emergency Team, 2020b). Second, among the healthcare workforce, there have been increased rates of infection ( Health Service Executive, 2020a; Health Service Executive, 2021c; Riley *et al.*, 2020) as well as altered behaviour patterns in terms of increased absences from work ( Gohar *et al.*, 2020; Nabe-Nielsen *et al.*, 2021). And third, altered patterns of GP referral and of patient self-referrals to hospital have emerged, which may impact hospital workloads and waiting lists ( Brick *et al.*, 2020; Heneghan & Jefferson, 2020; Lazzerini *et al.*, 2020; Marron *et al.*, 2021; NHS England, 2020).

This emerging body of literature has provided important insights into how the pandemic has affected healthcare activity in the Irish context, but, to date, the potential implications for healthcare reform have not been fully interrogated. As a consequence, this research focused on how we can harness the learnings from the Irish health system responses to COVID-19 to transform patient access, experience and outcomes as we start to think about the ‘bigger picture’ of implementing universal healthcare in Ireland.

This research is a component of a broader research project that sets out to examine the impact of COVID-19 on the Sláintecare implementation process (for a detailed overview of this study see ( Burke *et al.*, 2020)). In this paper, we draw on publicly available data to explore the effect of the pandemic on three key areas of care in the Irish context – primary care, community-based allied healthcare, and hospitals – during the first nine months of the pandemic. A core objective was to identify potential early lessons that may be relevant for the implementation of Sláintecare in 2021 and beyond.

## Methods

### Study design

Secondary analysis was undertaken on existing published and publicly available data that were: 1) related to primary care, community-based healthcare and hospital care in Ireland; 2) considered relevant for addressing this study’s research objectives; and 3) reported during the first nine months of the pandemic. Secondary analysis is a process whereby data collected by one or more researchers or administrative systems are re-analysed to pursue an alternative perspective on the same topic or a new research interest entirely.

### Data sources

1.   Primary care

There is a dearth of publicly available data on GP activity in Ireland. Prior to COVID-19, the need for a centralised data registry for GPs was recognised ( Health Service Executive, 2015; Walsh *et al.*, 2019). To describe GP activity nationally, this paper drew on selected data retrieved from:

   I.   The Primary Care Reimbursement Service (PCRS) ( Primary Care Reimbursement Service, 2021);
   II.   Health Service Executive (HSE) monthly Performance Reports ( Health Service Executive, 2018a; Health Service Executive, 2018b; Health Service Executive, 2018c; Health Service Executive, 2018d; Health Service Executive, 2018e; Health Service Executive, 2018f; Health Service Executive, 2018g; Health Service Executive, 2018h; Health Service Executive, 2018i; Health Service Executive, 2018j; Health Service Executive, 2018k; Health Service Executive, 2019a; Health Service Executive, 2019b; Health Service Executive, 2019c; Health Service Executive, 2019d; Health Service Executive, 2019e; Health Service Executive, 2019f; Health Service Executive, 2019g; Health Service Executive, 2019h; Health Service Executive, 2019i; Health Service Executive, 2019j; Health Service Executive, 2019k; Health Service Executive, 2019l; Health Service Executive, 2020b; Health Service Executive, 2020c; Health Service Executive, 2020d; Health Service Executive, 2020e; Health Service Executive, 2020f; Health Service Executive, 2020g; Health Service Executive, 2020h; Health Service Executive, 2020i; Health Service Executive, 2020j).
   III.   An open-access data source published by Ordnance Survey Ireland ( Ordnance Survey Ireland, 2021).

2.   Community-based healthcare

Waiting list and activity data were extrapolated from HSE monthly Performance Reports and quarterly Performance Profiles ( Health Service Executive, 2018a; Health Service Executive, 2018b; Health Service Executive, 2018c; Health Service Executive, 2018d; Health Service Executive, 2018e; Health Service Executive, 2018f; Health Service Executive, 2018g; Health Service Executive, 2018h; Health Service Executive, 2018i; Health Service Executive, 2018j; Health Service Executive, 2018k; Health Service Executive, 2018l; Health Service Executive, 2018m; Health Service Executive, 2018n; Health Service Executive, 2018o; Health Service Executive, 2019a; Health Service Executive, 2019b; Health Service Executive, 2019c; Health Service Executive, 2019d; Health Service Executive, 2019e; Health Service Executive, 2019f; Health Service Executive, 2019g; Health Service Executive, 2019h; Health Service Executive, 2019i; Health Service Executive, 2019j; Health Service Executive, 2019k; Health Service Executive, 2019l; Health Service Executive, 2019m; Health Service Executive, 2019n; Health Service Executive, 2019o; Health Service Executive, 2019p; Health Service Executive, 2020b; Health Service Executive, 2020c; Health Service Executive, 2020d; Health Service Executive, 2020e; Health Service Executive, 2020f; Health Service Executive, 2020g; Health Service Executive, 2020h; Health Service Executive, 2020i; Health Service Executive, 2020j; Health Service Executive, 2020k; Health Service Executive, 2020l; Health Service Executive, 2020m) to facilitate a comparison of the 2020 figures with those reported in 2018 and 2019. We present analysis on the following:

I. Eight community-based healthcare specialities, including:
  ∙ Physiotherapy
  ∙ Occupational therapy
  ∙ Speech and language therapy
  ∙ Audiology
  ∙ Psychology
  ∙ Podiatry
  ∙ Community ophthalmology
  ∙ Dietetics
II. Infants receiving developmental screening checks at 10 months of age from community-based public health nurses (PHNs) ( Health Service Executive, 2018l; Health Service Executive, 2018m; Health Service Executive, 2018n; Health Service Executive, 2018o; Health Service Executive, 2019m; Health Service Executive, 2019n; Health Service Executive, 2019o; Health Service Executive, 2019p; Health Service Executive, 2020k; Health Service Executive, 2020l; Health Service Executive, 2020m).

It is important to note that in both cases, these figures relate only to the public (State-run) health services. To this end, data on access, wait times and activity levels in the private sector remain unavailable and thus excluded from this research.

3.   Hospital care

Hospital waiting list data were extracted from reports published by the National Treatment Purchase Fund (NTPF) ( National Treatment Purchase Fund, 2021). There are three major waiting list categories defined by the NTPF Waiting List Management Protocol ( National Treatment Purchase Fund, 2017):

I. Outpatient Waiting Lists. This category refers to patients awaiting their first appointment at a consultant-led outpatient clinic.
II. In-patient/day-cases (IPDCs). This category refers to patients awaiting admission on an elective basis for care or treatment. Many surgical procedures fall into this category, such as elective hip or knee replacements, as well as investigative procedures such as gastrointestinal (GI) endoscopy. ‘In-patient’ admissions refer to those patients who will require use of a hospital bed overnight following their treatment, while ‘day-case’ admissions refer to those discharged home on the same day.
III. Planned procedures (PPs). This category refers to patients who have already had an initial episode of care and require further treatment. Common day-case PPs include second-eye cataract surgery, skin grafts, and follow-up GI endoscopy.

Within each of these categories, hospital waiting lists are subdivided into ‘active’ (those awaiting a treatment appointment date), ‘suspended’ (those who are ‘temporarily unfit or unable to attend due to clinical or personal/social reasons’), and ‘to come in (TCI)’ (those who have received an appointment for their treatment) (see Table 3). The complexity of these numerous subcategories creates difficulty both in capturing the full burden of patient wait times and in enabling comparisons with other jurisdictions ( Brick & Connolly, 2021).

### Analysis

Once these selected data were extracted, cleaned and prepared for analysis, descriptive statistics were computed using Microsoft Excel 2016.

## Results

### Primary care

**Figure 1.**
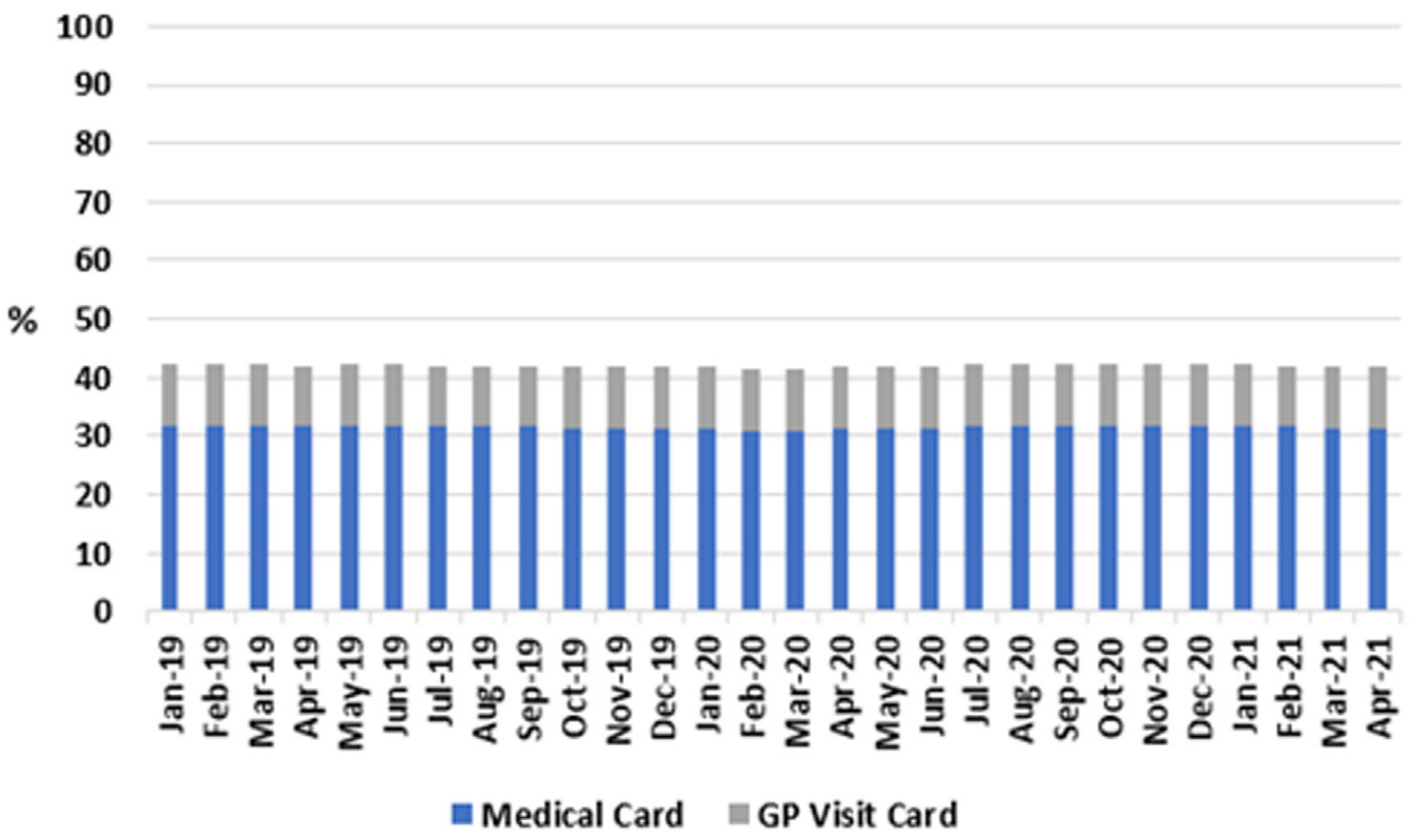
Share of population eligible for either medical card or GP Visit Card, per month (2018-2021) (from Primary Care Reimbursement Service).

***Eligibility categories***. As shown in Figure 1, the proportion of the Irish population eligible for free-at-point-of-use GP care, due to their medical card or GP Visit Card, was analysed from PCRS data for 2019, 2020, and the months of 2021 for which data were available.

For all months studied, the proportions of the population eligible for a GP visit card or a medical card remained stable, between 10.3-10.6% and 31.0-31.9% respectively. Accordingly, the proportion of the population entitled to GP care without charge remained between 41.6% and 42.4% across the study period, including after onset of the pandemic in 2020.

**Figure 2.**
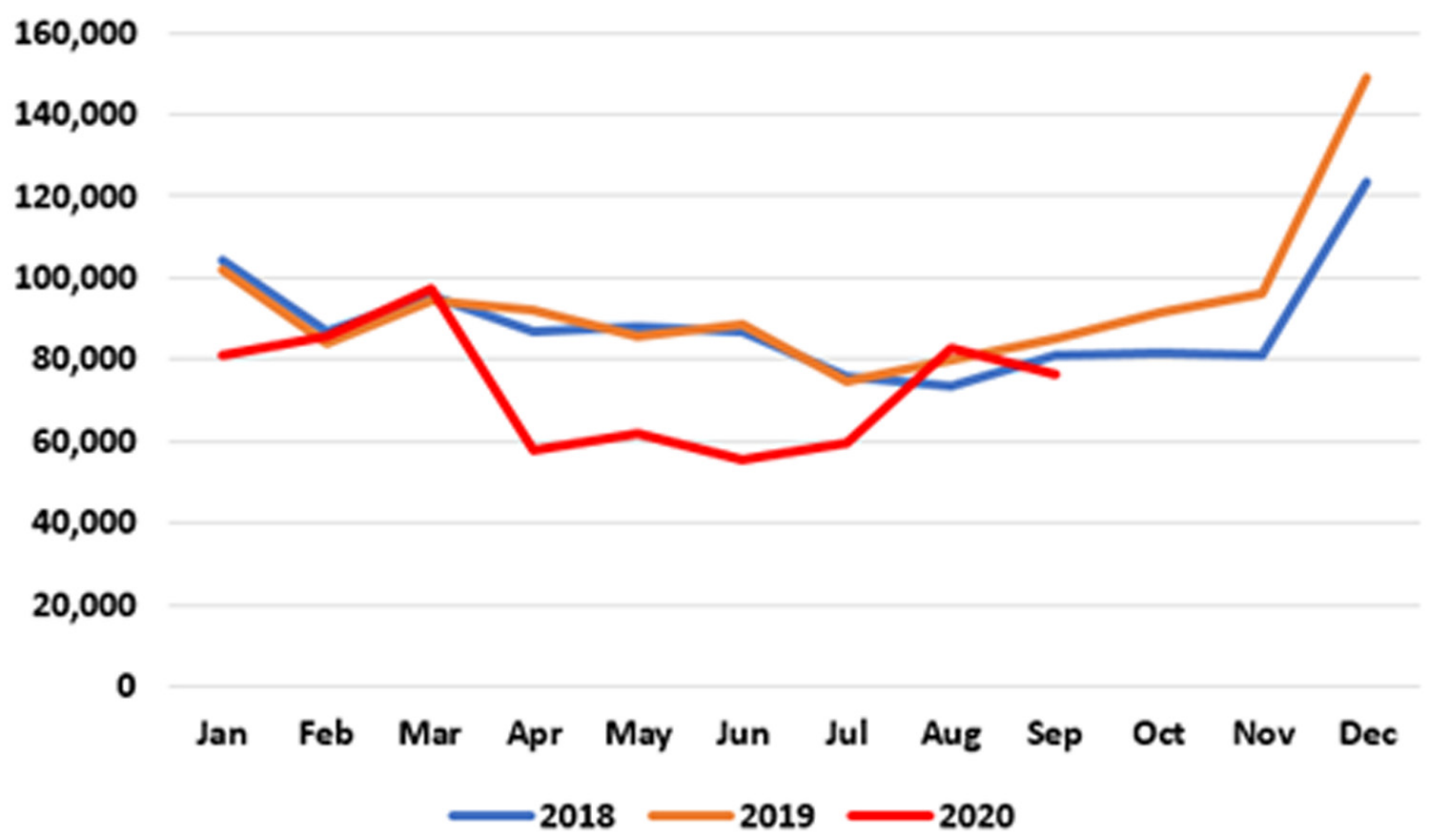
Monthly use of GP out-of-hours services (2018-2020) (from HSE Performance Reports).

***Out-of-hours GP care***. Data on patients accessing out-of-hours (OOH) GP care are published at monthly intervals. As seen in Figure 2, the number of patients availing of OOH GP care in the early months of 2020 is similar to those seen in 2018 and 2019. However, a sharp decline emerged in April 2020, when 57,945 patients were seen nationally by an OOH GP service compared to 92,369 in April 2019 – a reduction of 37.3%, or 34,424. This decline was maintained until July 2020. In August and September 2020, OOH GP care activities returned to pre-pandemic levels.

It should be noted that these data may under-state the full extent of activity as reported to the HSE, particularly for private patients paying out of pocket at the time of the consultation.

**Figure 3.**
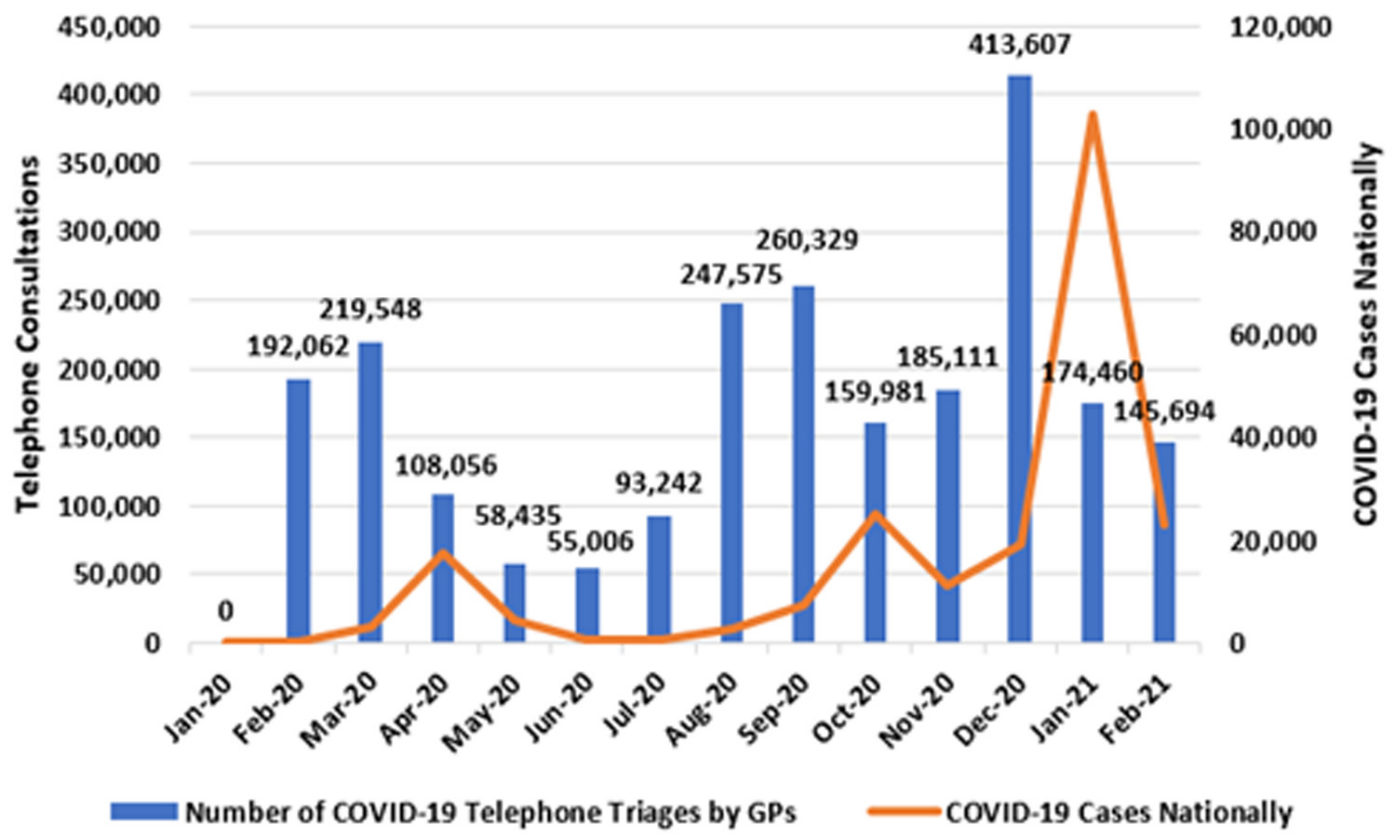
Number of COVID-19 telephone triages by GPs (running totals shown) with national COVID-19 cases (2020-2021) (from Primary Care Reimbursement Service).

***COVID-19 telephone consultations***. Since the onset of the pandemic, GPs have been reimbursed by the HSE via the PCRS to conduct telephone-based triaging consultations with patients with possible COVID-19 symptoms. These consultations are provided without financial charges for patients. Figure 3 presents the monthly number of telephone triages at the national level (left axis), alongside national case totals (right axis).

As shown, there were three distinct peaks in COVID-19 telephone consultation activity by GPs: in March, September and December 2020. These peaks preceded the national peaks in COVID-19 cases, seen in April 2020, October 2020, and January 2021, respectively.

In March 2020, the ‘spring peak’ manifested as 219,548 telephone consultations by GPs triaging COVID-19 symptoms nationally. Activity level then fell during the summer months, before reaching the ‘autumn peak’ of 260,329 COVID-19 telephone triages in September 2020. This was exceeded in December 2020, when 416,607 telephone triages occurred. This high-point preceded the surge in case numbers seen in January 2021, when 103,015 new cases of COVID-19 were identified and the national incidence was temporarily the highest in the world ( Ritchie *et al.*, 2020). In total, from February 2020 to February 2021, 2.31 million of these consultations occurred.

**Figure 4.**
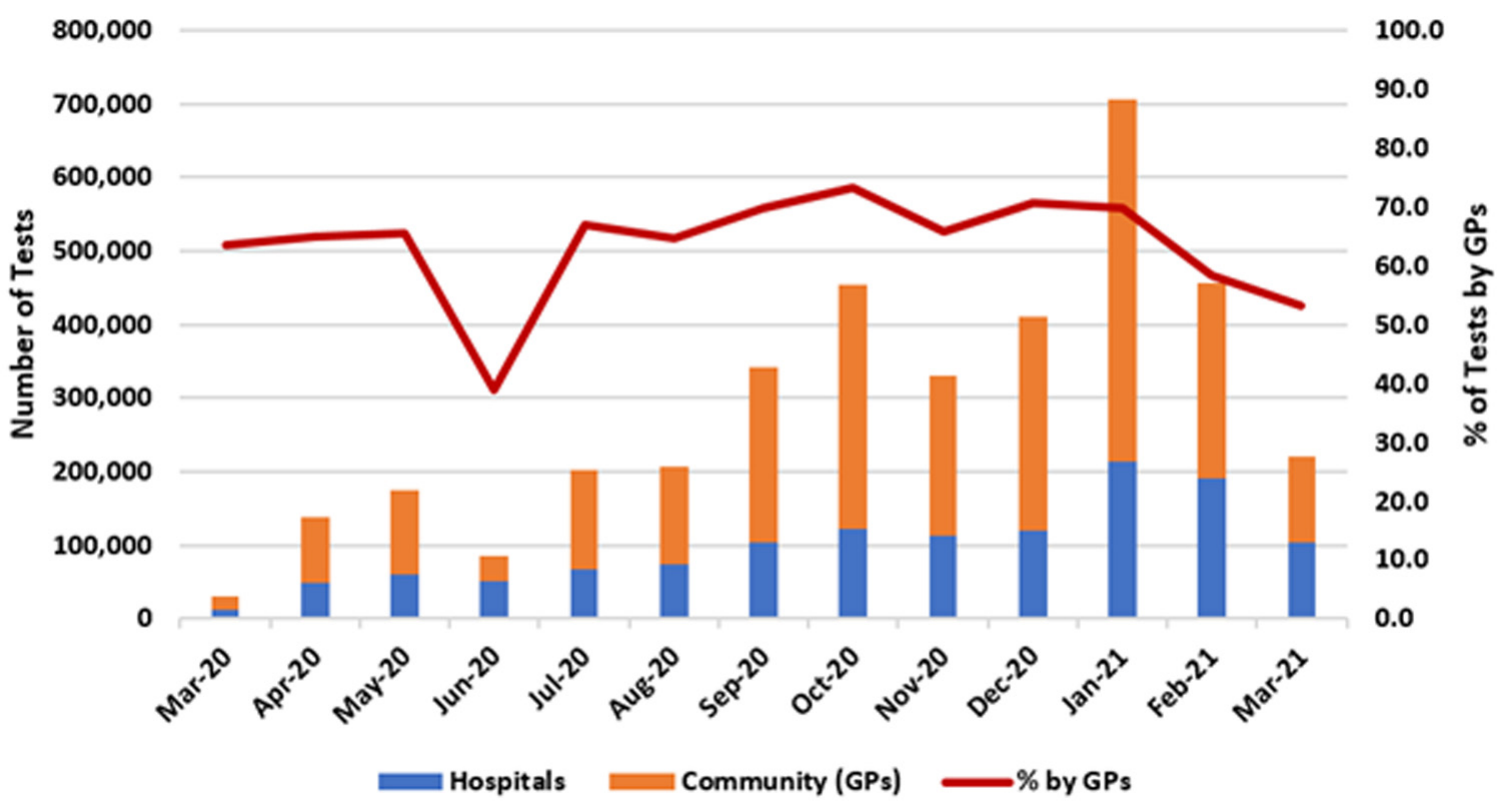
COVID-19 testing nationally by location (hospital or community/GPs) with percentage of tests from community/GPs (from Ordnance Survey Ireland open-source data).

***COVID-19 testing by GPs***. Figure 4 presents the number of COVID-19 PCR tests conducted for the hospital sector and the community.

As of March 2021, 3.76 million COVID-19 tests were performed by Ireland’s public healthcare system, 2.48 million (66%) of which were referred from the community. The largest monthly number of COVID-19 tests was seen in January 2021, with 705,531 performed, of which 491,772 (69.7%) arose from the community.

As shown by the red line in Figure 4, the proportion of tests in which GPs played at least some role – that is, ordering them, following up on them, or both – was between 60% and 70% for most months of the pandemic, averaging 66%, with a peak of 73.8% in October 2020.

Of note, for the purposes of this study and in the absence of more robust data, it is assumed that COVID-19 tests performed in hospitals were ordered by a hospital-based clinician, while those performed outside of hospitals were either ordered by GPs, followed up by them, or both. However, a small proportion of out-of-hospital COVID-19 tests may have originated from other sources, such as from public health doctors referring close contacts of confirmed cases for testing, as part of a response to a specific outbreak or cluster. As such, these data may over-state GPs’ role in referrals for testing.

In addition, these data do not include COVID-19 tests performed in the private sector or in ‘walk-in’ testing centres which began to operate in early 2021.

### Community-based healthcare specialties

***Activity levels***. As mentioned earlier, analysis on activity levels and waiting lists was undertaken using data on eight community-based healthcare specialties, including: physiotherapy; occupational therapy; speech and language therapy; audiology; psychology; podiatry; community ophthalmology; and dietetics.

**Figure 5.**
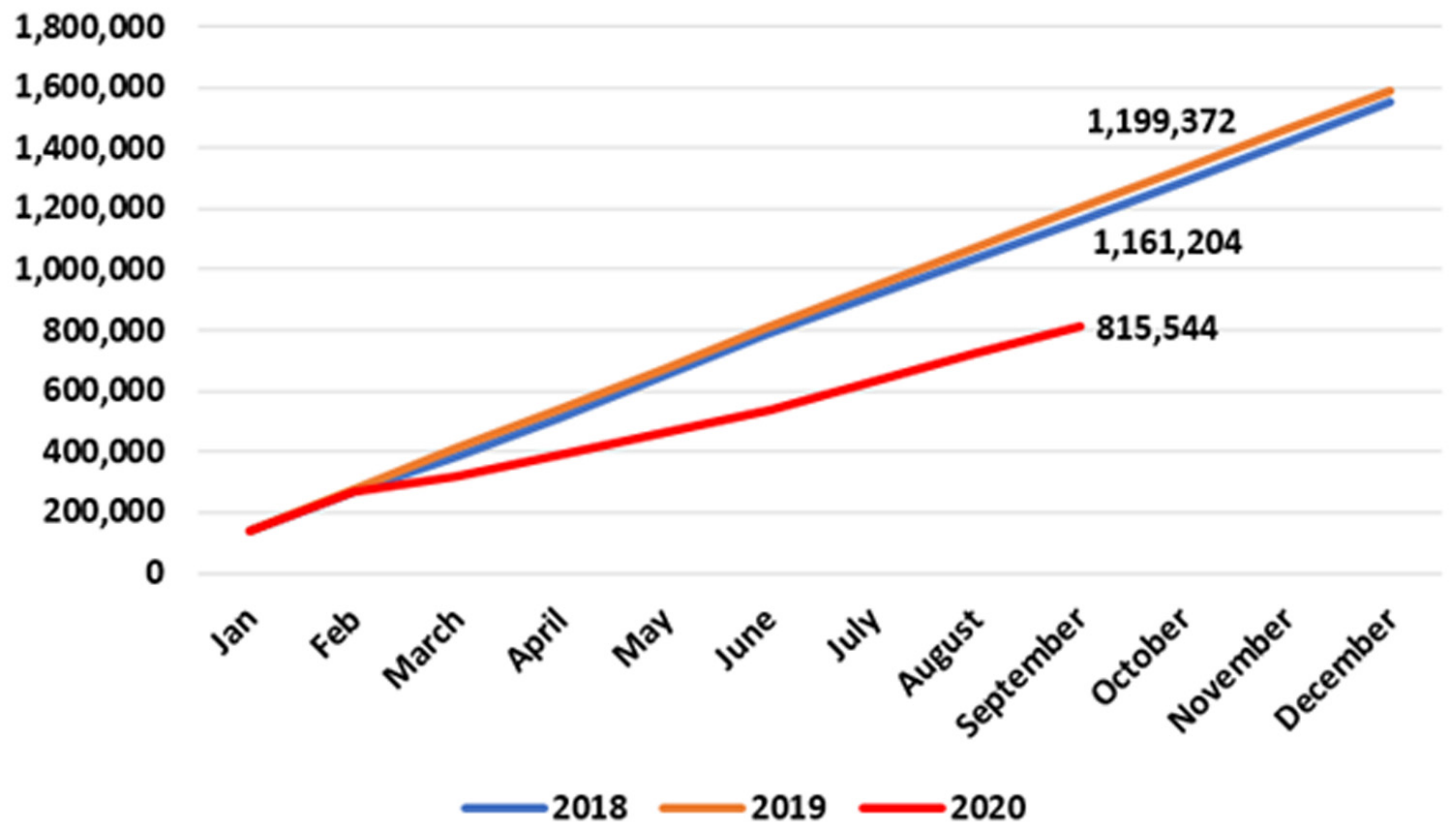
Cumulative patient numbers seen across eight allied healthcare specialties. Totals for September 2018 (middle figure), 2019 (top) and 2020 (bottom) are labelled. (from HSE Performance Reports).

As shown in Figure 5, there was a 35% reduction in publicly provided care across all eight allied health specialties in the community when the first nine months of 2020 are compared to the same period in 2018 and 2019, suggesting a significant impact of COVID-19 on these essential services. The divergence appeared in March 2020, when the HSE introduced widespread restrictions on non-urgent healthcare.

By September 2020, 771,322 patients were seen by all eight specialties, compared to 1,157,154 and 1,187,734 patients by the same date in 2018 and 2019, respectively. This 2020 figure represents a reduction of 416,412 (35.1%) compared to 2019 (data for the last three months of 2020 are unavailable at time of writing).

There was heterogeneity observed between specialties regarding the degree of reduction of activity seen in 2020 when compared to the 2019 figures (see Table 1). The greatest reductions in patient numbers were seen in speech and language therapy (49.0%) and audiology (46.1%), while dietetics (13.6%) and psychology (18.1%) exhibited the lowest proportional increase over the observation period.

**Table 1.** Patients seen in eight community-based specialties on a cumulative basis (year-to-date (YTD) to September, 2018-2020) (from HSE Performance Reports).

	September 2018 YTD	September 2019 YTD	September 2020 YTD	2020 vs. 2019	
				Absolute reduction	% Difference
Physiotherapy	430,424	427,806	283,745	144,061	-33.7
Occupational Therapy	264,591	286,572	207,944	78,628	-27.4
Speech & Language Therapy	209,010	207,710	105,878	101,832	-49.0
Psychology	31,201	33,900	27,751	6,149	-18.1
Podiatry	62,360	62,181	36,910	25,271	-40.6
Community Ophthalmology	74,154	75,809	41,564	34,245	-45.2
Audiology	38,004	41,473	22,368	19,105	-46.1
Dietetics	47,410	52,283	45,162	7,121	-13.6
All Specialties	1,157,154	1,187,734	771,322	416,412	-35.1

**Figure 6.**
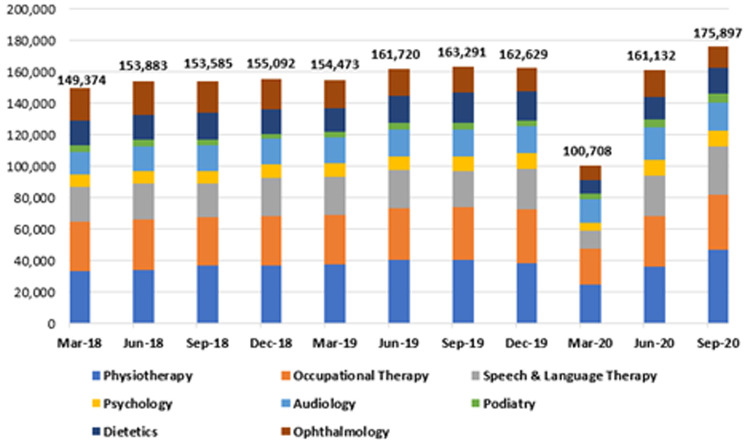
Total waiting list figures across all eight community-based specialties (2018–2020) (from HSE Performance Reports).

***Community waiting lists***. Waiting lists for the eight community specialties were also analysed at three-month intervals from 2018 to 2020. These are represented in Table 2 and Figure 6. As shown, this period was characterised by three phases. First, there was a gradual, sustained increase in waiting list numbers across the specialties in 2018 and 2019. Second, an abrupt decline in the waiting list numbers for all specialties combined was reported in the first quarter of 2020, falling from 162,629 in December 2019 to 100,708 in March 2020, a reduction of 61,921 or 38.1%. Thirdly, a marked increase was seen in waiting list numbers from March to September 2020 for all specialties, offsetting the decline seen earlier that year.

**Table 2.** Waiting list numbers for eight community specialties (2018-2020), with proportions waiting more than one year (from HSE Performance Reports).

	Physiotherapy		Occupational Therapy		Speech & Language Therapy		Psychology	
	Total	% Waiting >1yr	Total	% Waiting >1yr	Total	% Waiting >1yr	Total	% Waiting >1yr
Mar-18	33,358	5.0	31,296	22.8	21,956	5.2	8,142	25.5
Jun-18	34,161	5.2	31,934	23.6	22,924	5.7	8,055	24.1
Sep-18	36,664	6.0	30,880	25.1	21,373	7.1	7,733	23.6
Dec-18	36,706	5.7	31,867	25.6	24,115	7.4	8,087	24.2
Mar-19	37,392	5.9	31,776	26.8	23,858	6.9	8,820	23.8
Jun-19	40,749	6.1	32,888	26.7	23,660	14.2	8,498	24.5
Sep-19	40,794	6.9	33,434	27.8	22,368	10.9	9,276	28.4
Dec-19	38,177	8.0	34,343	29.2	25,749	11.8	10,092	32.3
Mar-20	25,063	12.4	22,591	29.0	11,129	13.3	5,456	33.7
Jun-20	36,431	17.1	31,877	36.5	25,780	20.8	9,757	41.3
Sep-20	47,136	18.4	34,658	38.9	30,810	25.6	10,135	47.1
	Podiatry		Community Ophthalmology		Audiology		Dietetics	
	Total	% Waiting >1yr	Total	% Waiting >1yr	Total	% Waiting >1yr	Total	% Waiting >1yr
Mar-18	3,972	22.0	20,707	41.5	14,326	13.8	15,617	30.4
Jun-18	3,752	25.6	21,149	41.0	15,740	13.3	16,168	30.5
Sep-18	3,594	26.6	19,411	41.0	16,431	13.9	17,499	29.5
Dec-18	3,174	31.0	18,806	38.8	16,692	13.7	15,645	22.3
Mar-19	3,621	28.9	17,850	35.7	16,193	12.9	14,963	21.5
Jun-19	3,900	21.6	17,044	33.4	17,621	13.5	17,360	19.4
Sep-19	4,001	24.6	16,690	31.8	17,487	15.1	19,241	19.5
Dec-19	3,504	27.7	15,119	32.9	17,110	16.4	18,535	22.9
Mar-20	3,365	30.0	9,833	34.3	14,773	19.3	8,498	18.5
Jun-20	4,633	35.5	17,425	37.4	21,136	27.3	14,093	33.4
Sep-20	5,800	40.4	13,104	38.8	17,661	38.3	16,593	32.3

As shown in Table 2, while waiting lists for all specialties increased markedly in the period from March to September 2020, speech and language therapy and dietetics recorded the greatest increases during this time, rising by 176.8% (11,129 to 30,810) and 95.3% (8,498 to 16,593), respectively.

(Of note, distinct waiting lists for assessment and treatment are published by the HSE for speech and language therapy [ Health Service Executive, 2018a; Health Service Executive, 2018b; Health Service Executive, 2018c; Health Service Executive, 2018d; Health Service Executive, 2018e; Health Service Executive, 2018f; Health Service Executive, 2018g; Health Service Executive, 2018h; Health Service Executive, 2018i; Health Service Executive, 2018j; Health Service Executive, 2018k; Health Service Executive, 2018l; Health Service Executive, 2018m; Health Service Executive, 2018n; Health Service Executive, 2018o; Health Service Executive, 2019a; Health Service Executive, 2019b; Health Service Executive, 2019c; Health Service Executive, 2019d; Health Service Executive, 2019e; Health Service Executive, 2019f; Health Service Executive, 2019g; Health Service Executive, 2019h; Health Service Executive, 2019i; Health Service Executive, 2019j; Health Service Executive, 2019k; Health Service Executive, 2019l; Health Service Executive, 2019m; Health Service Executive, 2019n; Health Service Executive, 2019o; Health Service Executive, 2019p; Health Service Executive, 2020b; Health Service Executive, 2020c; Health Service Executive, 2020d; Health Service Executive, 2020e; Health Service Executive, 2020f; Health Service Executive, 2020g; Health Service Executive, 2020h; Health Service Executive, 2020i; Health Service Executive, 2020j; Health Service Executive, 2020k; Health Service Executive, 2020l; Health Service Executive, 2020m]; these are combined in this section.)

**Figure 7.**
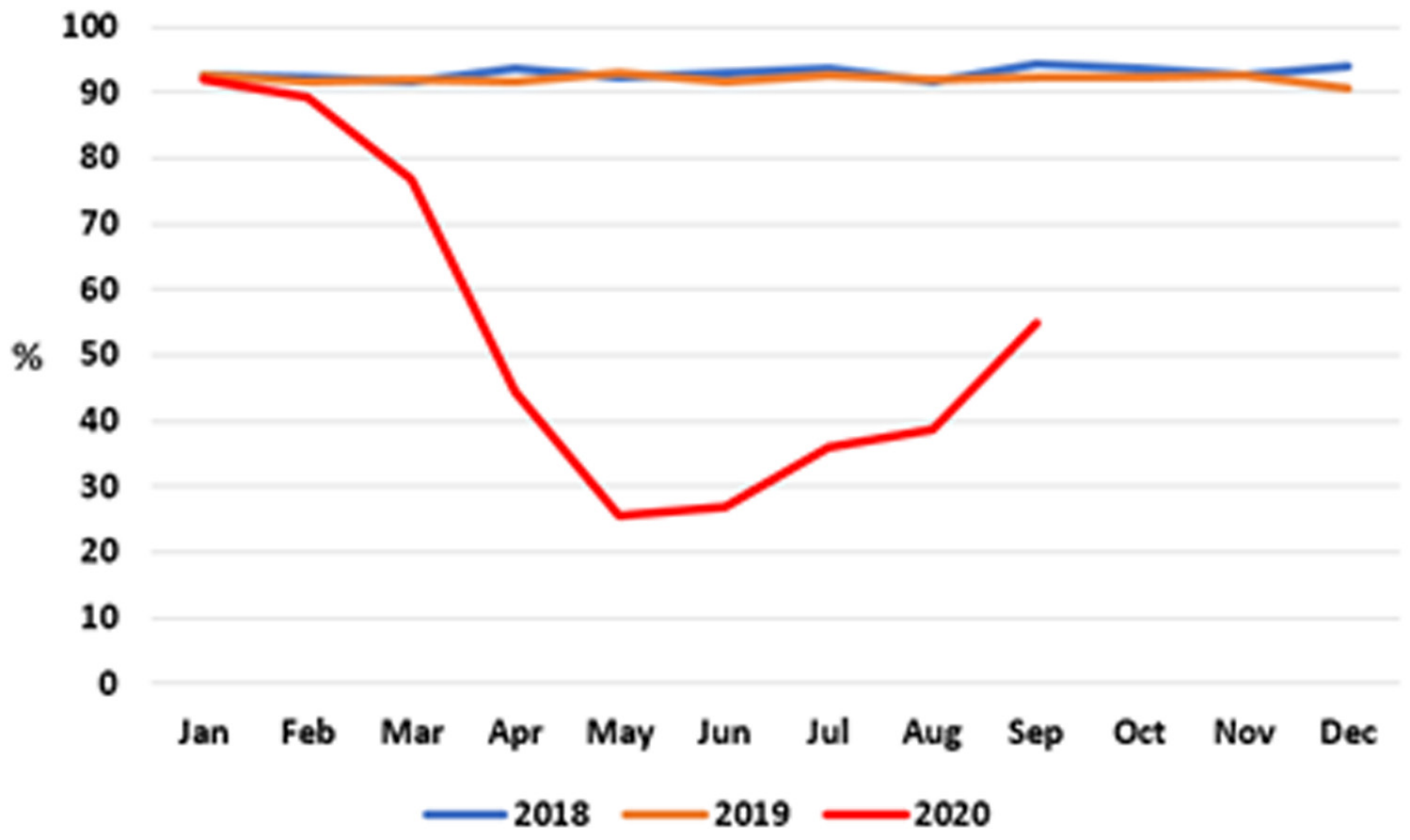
Percentage of infants receiving PHN 10-month developmental screening check on time (2018–2020) (from HSE Performance Reports).

***Developmental screening checks***. The proportion of infants receiving developmental screening checks on time – that is, by 10 months of age – from a PHN is shown at monthly intervals in Figure 7 (data after September 2020 were unavailable at time of writing).

As can be seen, throughout 2018 and 2019, the proportion of children receiving this screening check on time was consistently between 90% and 94%. There was a marked decline in achieving this target in the first half of 2020, reaching a nadir of 25.5% in May 2020 before recovering slightly to 54.8% in September 2020, the latest month for which data are available.

### Hospital care

**Figure 8.**
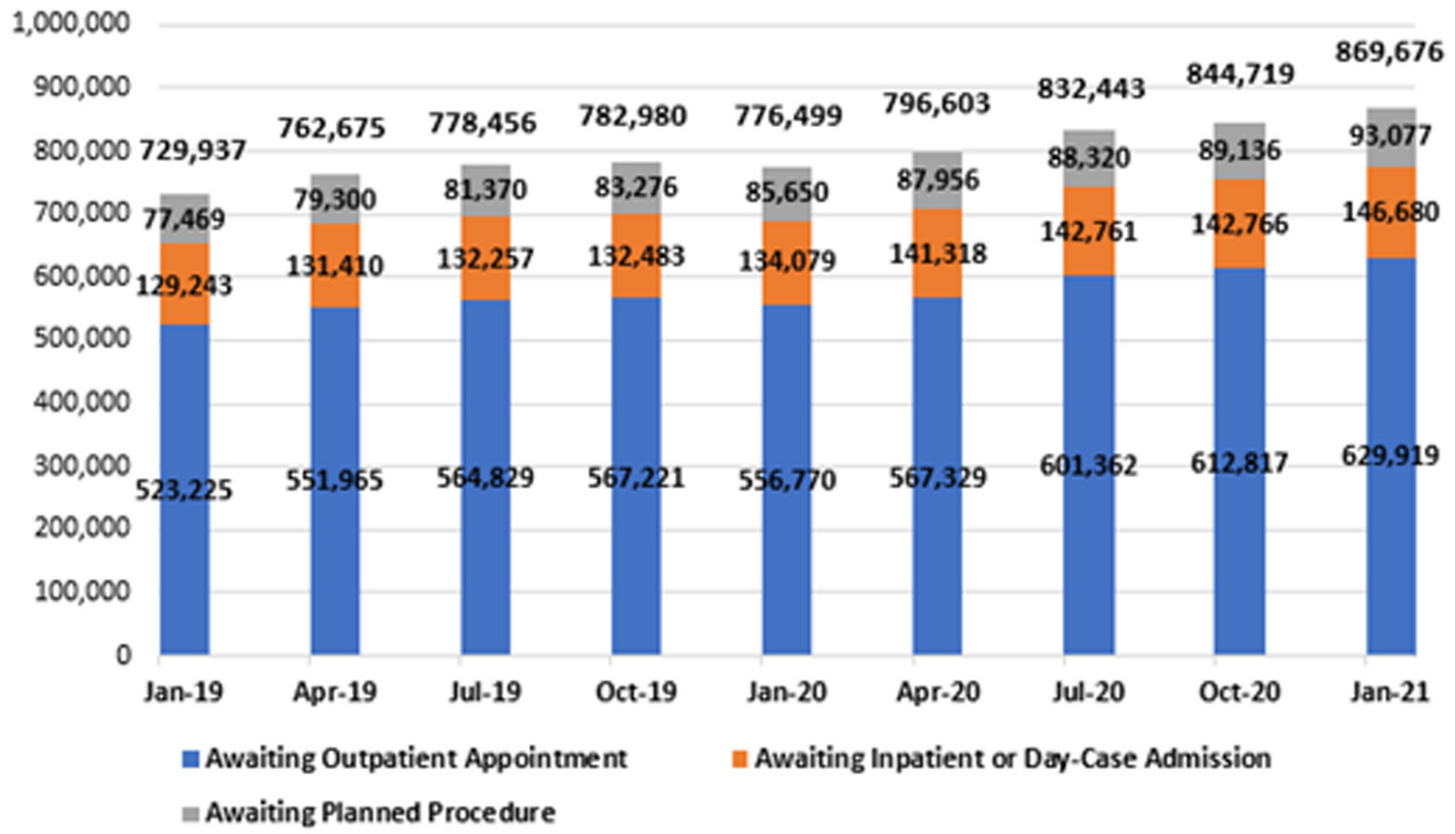
National hospital waiting list numbers from January 2019 to January 2021, by category (from National Treatment Purchase Fund).

In January 2021, there were 869,676 people waiting on some form of Irish public hospital waiting list across all NTPF waiting list categories, as shown in Figure 8 and in greater detail in Table 3. This total refers to the number of patients across the three major hospital waiting list categories – outpatients, in-patient/day-case admissions and planned procedures – including those grouped as ‘TCI’ or ‘suspended’.

**Table 3.** Overview of all waiting list categories at three-monthly intervals from January 2019 to January 2021 ( National Treatment Purchase Fund, 2021).

	Jan-19	Apr-19	Jul-19	Oct-19	Jan-20	Apr-20	Jul-20	Oct-20	Jan-21
**Category (i) – Outpatients**									
Active	523,225	551,965	564,829	567,221	556,770	567,329	601,362	612,817	622,963
Suspended	0	0	0	0	0	0	0	0	6,956
Category (i) Total	523,225	551,965	564,829	567,221	556,770	567,329	601,362	612,817	629,919
**Category (ii) – Inpatient/Day-Cases**									
Active	72,027	70,295	68,807	67,511	67,303	86,343	80,283	74,860	81,456
‘To Come In’	17,975	19,289	19,886	19,649	20,033	5,806	11,377	14,745	8,088
Suspended	7,427	7,344	8,542	9,879	10,984	8,508	7,637	8,711	11,607
Subtotal – Excluding Endoscopy	97,429	96,928	97,235	97,039	98,320	100,657	99,297	98,316	101,151
GI Endoscopy, Active	19,748	22,220	22,592	21,979	22,231	34,110	34,983	34,116	36,065
GI Endoscopy, ‘To Come In’	9,335	9,810	9,608	9,275	8,882	1,732	5,045	6,143	3,919
GI Endoscopy, Suspended	2,731	2,452	2,822	4,190	4,646	4,819	3,436	4,191	5,545
Subtotal – GI Endoscopy Only	31,814	34,482	35,022	35,444	35,759	40,661	43,464	44,450	45,529
Category (ii) Total	129,243	131,410	132,257	132,483	134,079	141,318	142,761	142,766	146,680
**Category (iii) - Planned Procedures**									
Active	13,815	14,170	14,735	14,777	15,202	15,092	14,749	14,809	16,881
Suspended	82	79	110	155	108	203	117	,99	220
Subtotal – Excluding Endoscopy	13,897	14,249	14,845	14,932	15,310	15,295	14,866	14,908	17,101
GI Endoscopy, Active	61,360	62,882	64,154	65,992	68,042	70,320	71,238	72,295	73,991
GI Endoscopy, Suspended	2,212	2,169	2,371	2,352	2,298	2,341	2,216	1,933	1,985
Subtotal – GI Endoscopy Only	63,572	65,051	66,525	68,344	70,340	72,661	73,454	74,228	75,976
Category (iii) Total	77,469	79,300	81,370	83,276	85,650	87,956	88,320	89,136	93,077
**Total – All Categories**	**729,937**	**762,675**	**778,456**	**782,980**	**776,499**	**796,603**	**832,443**	**844,719**	**869,676**

All three categories underwent an increase during this two-year period. Outpatient waiting lists increased by 20.4%, reaching 629,919 in January 2021; in-patient and day-case waiting lists increased by 13.5%, reaching 146,680; and planned procedure waiting lists increased by 20.1%, reaching 93,077. In total, the national hospital waiting list figure was 139,739 in January 2021, 19.1% higher than two years prior.

**Figure 9.**
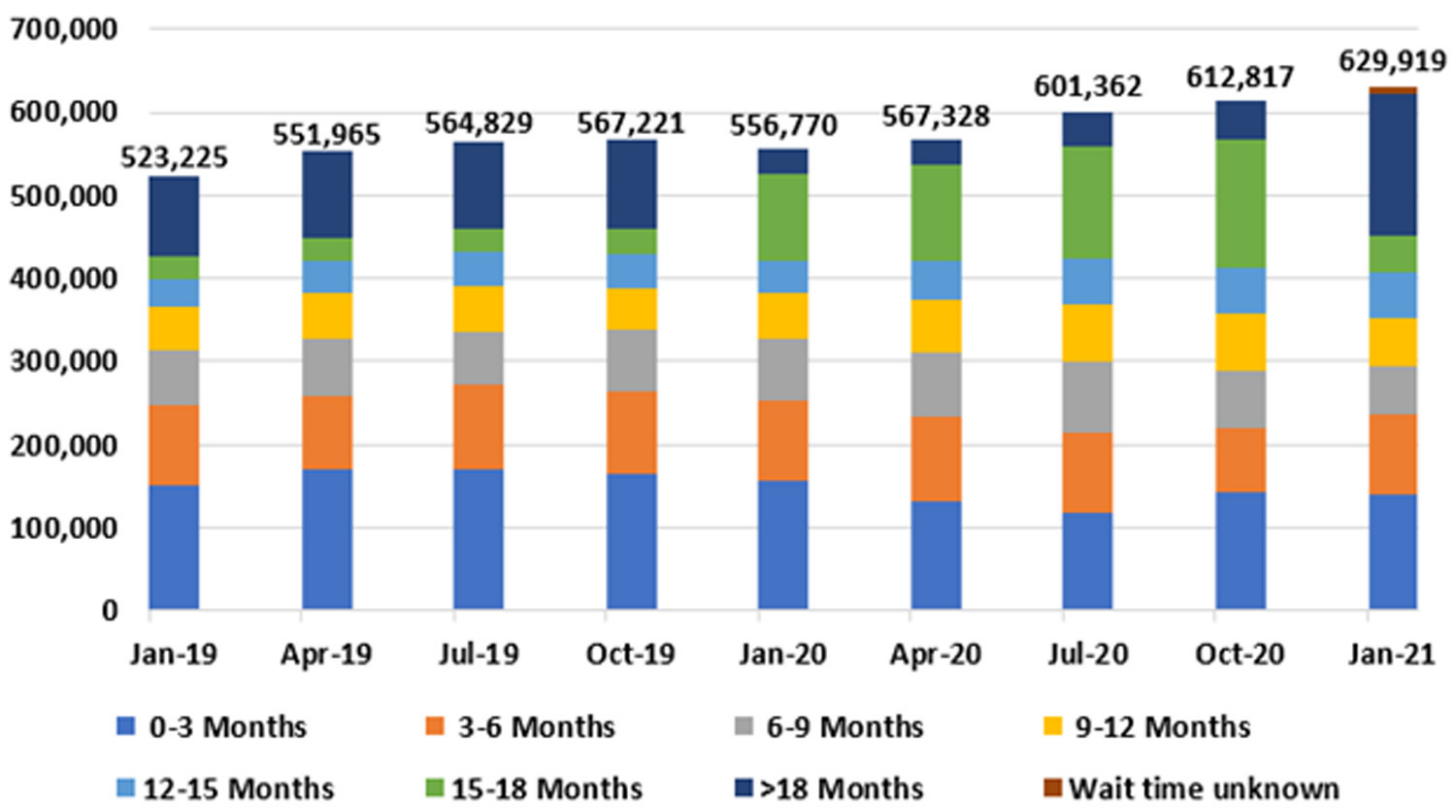
National outpatient waiting list figures, by length of wait (January 2019 - January 2021) (from National Treatment Purchase Fund).

***Outpatient waiting lists***. Figure 9 and Table 4 present national outpatient hospital waiting list data at three-month intervals from January 2019 to 2021, by length of wait. Most notably, outpatient waiting list numbers rose nationally from 523,225 in January 2019 to 629,919 in January 2021, representing an increase of 106,694, or 20.4%.

**Table 4.** Outpatient waiting lists at three-month intervals from January 2019 to 2021, by length of wait ( National Treatment Purchase Fund, 2021).

Wait Time	Jan-19	Apr-19	Jul-19	Oct-19	Jan-20	Apr-20	Jul-20	Oct-20	Jan-21
0-3 months	151,663	171,205	170,464	164,038	155,906	131,282	118,741	143,284	140,711
3-6 months	94,400	87,866	101,849	100,367	97,487	102,641	95,148	75,002	95,109
6-9 months	68,405	68,743	63,818	74,234	74,285	77,245	84,497	70,717	58,278
9-12 months	50,344	54,572	53,178	50,262	55,836	62,050	69,531	68,714	57,305
12-15 months	33,819	38,113	41,699	39,634	36,296	46,378	54,403	55,554	54,632
15-18 months	28,351	27,493	29,576	32,226	107,040	117,090	137,160	153,872	45,945
>18 months	96,243	103,973	104,245	106,460	29,920	30,642	41,882	45,674	170,983
Wait time unknown	0	0	0	0	0	0	0	0	6,956
Total	523,225	551,965	564,829	567,221	556,770	567,328	601,362	612,817	629,919

In addition, the proportion of patients experiencing very long waits for care increased during this time, with 170,983 (27.1%) of those on the outpatient waiting list waiting longer than 18 months as of January 2021 ( Table 5). The hospital specialties with the longest outpatient waiting lists in January 2021 were orthopaedic surgery (77,257); ear, nose and throat surgery (68,073); and ophthalmology (47,075), comprising 30.5% of the total national outpatient waiting list ( Table 6).

**Table 5.** Patients waiting longer than 18 months for a first hospital outpatient appointment (2019-2021) ( National Treatment Purchase Fund, 2021).

	Number waiting <18 months	% of OPD list
January 2019	96,243	18.4
April 2019	103,973	18.8
July 2019	104,245	18.5
October 2019	106,460	18.8
January 2020	29,920	5.4
April 2020	30,642	5.4
July 2020	41,882	7.0
October 2020	45,674	7.5
January 2021	170,983	27.1

**Table 6.** Two-year trends of the ten hospital specialties with longest outpatient waiting lists ( National Treatment Purchase Fund, 2021)

	Jan-19	Jan-20	Jan-21	% Change (2019 to 2021)
Orthopaedics	64,789	64,907	77,257	+19.2
ENT	67,520	64,229	68,073	+0.8
Ophthalmology	40,443	40,777	47,075	+16.4
Dermatology	43,544	43,363	46,010	+5.7
General Surgery	31,572	32,860	43,506	+37.8
Paediatrics	30,720	44,116	43,430	+41.4
Urology	29,670	30,911	33,528	+13
Gynaecology	28,029	27,220	30,180	+7.7
Cardiology	20,142	26,378	29,034	+44.1
Neurology	20,950	22,260	22,501	+7.4

**Figure 10.**
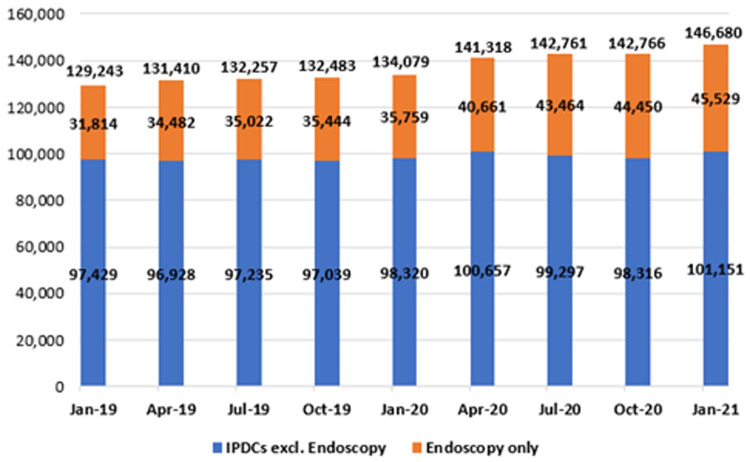
Number of patients on in-patient/day-case (IPDC) waiting lists (January 2019 - January 2021) (from National Treatment Purchase Fund).

**Figure 11.**
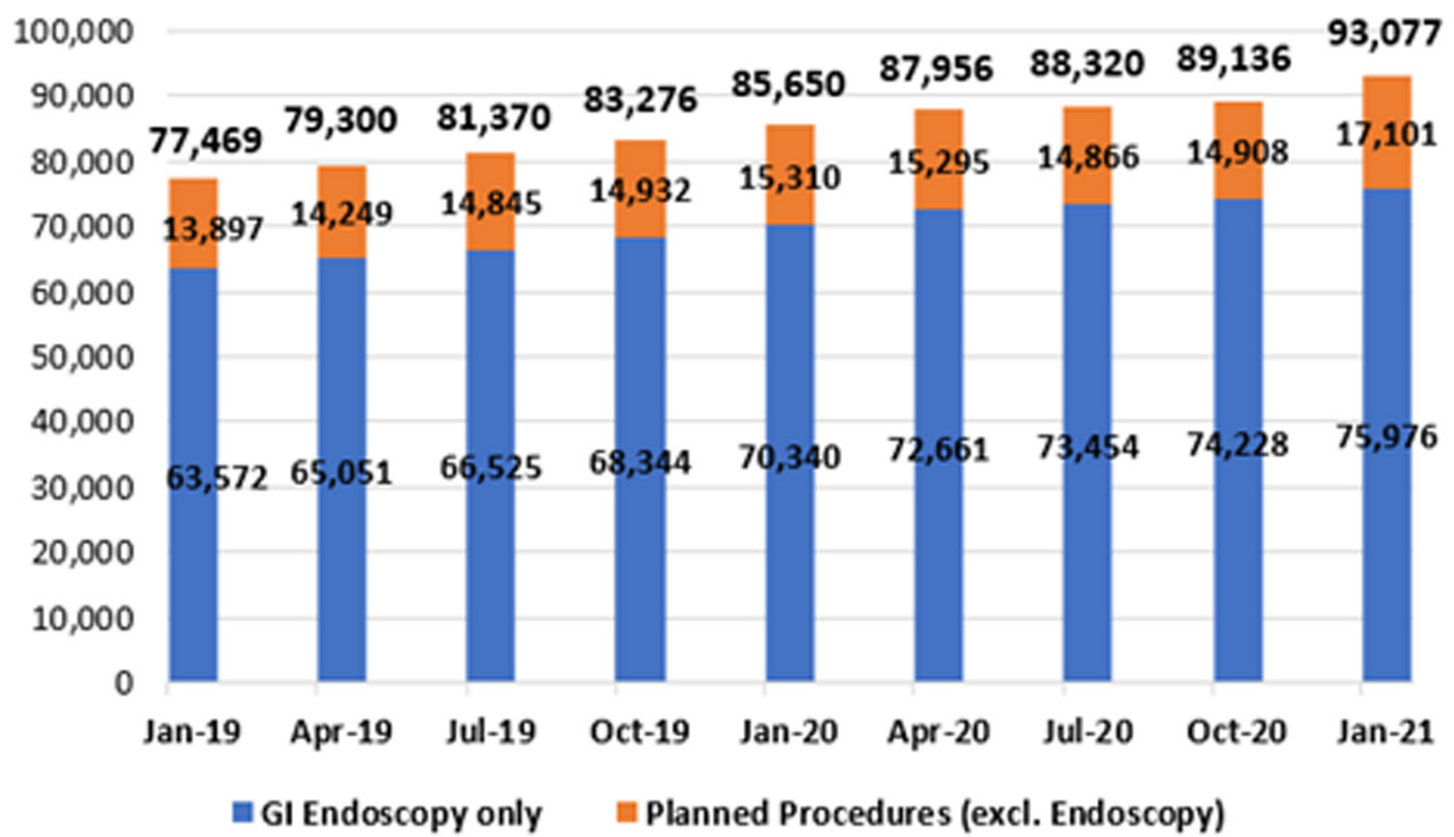
Planned procedure waiting lists (January 2019 – January 2021) (from National Treatment Purchase Fund).

***In-patient/day-case waiting lists***. Waiting lists for the IPDC admissions were analysed with respect to length of wait and are presented in Figure 10. As stated, this category refers to patients awaiting admission on an elective basis for care or treatment, some of whom require overnight admission after their treatment (in-patient admissions), together with those who do not (day-cases). Of note, NTPF figures for the IPDC and planned procedure waiting list categories are published with patients awaiting endoscopy considered separately; as shown in Figure 10 and Figure 11, data in this paper are depicted in the same manner.

As shown, there was a marked increase in both endoscopic and general IPDC waiting lists in the early months of 2020 (January to April), coinciding with the ‘first wave’ of COVID-19 and attendant disruptions in planned routine healthcare. From January 2019 to 2020, the number awaiting an IPDC admission rose by 3.7% from 129,243 to 134,079; from January 2020 to 2021, it rose by 9.4% from 134,079 to 146,680. As of January 2021, the three specialties with the largest IPDC waiting lists were general surgery (13,537 waiting), orthopaedic surgery (11,092) and urology (10,348).

***Planned procedures***. As mentioned earlier, planned procedures refer to those patients who have had an initial episode of care and require recall for further treatment subsequent to that episode. Patients in this category include those awaiting second-eye cataract surgery, follow-up skin grafts, or follow-up GI endoscopy. As shown in Figure 11, patients awaiting GI endoscopy constitute the majority of this category.

**Figure 12.**
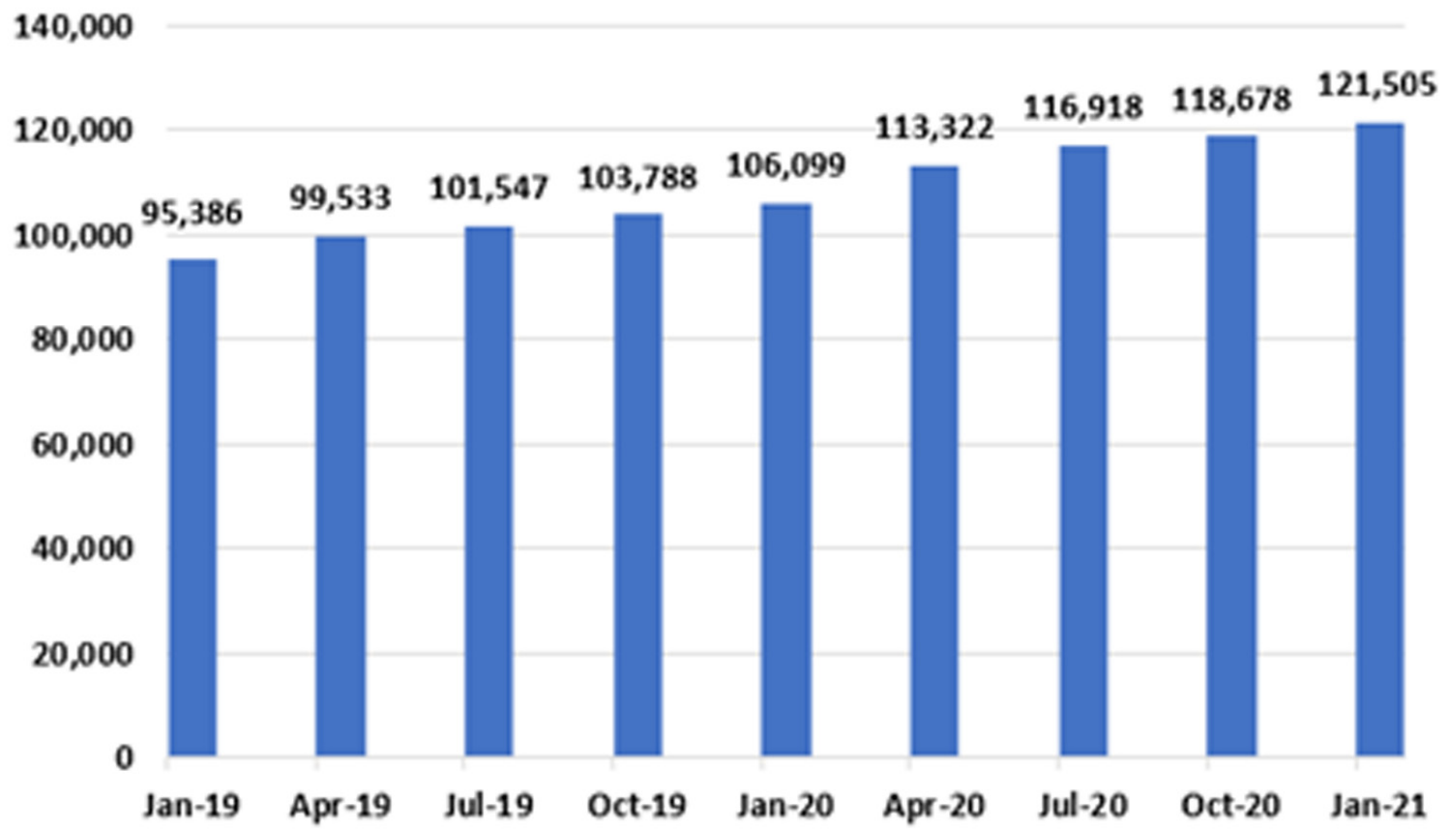
Total GI endoscopy waiting list figures in Ireland (January 2019 – January 2021) (from National Treatment Purchase Fund).

***Endoscopy***. Notably, as shown in Figure 10 and Figure 11, both the IPDC and planned procedure waiting list categories include a cohort of patients awaiting GI endoscopy. These cohorts are combined in Figure 12, to provide an estimate of total national patient numbers awaiting GI endoscopy.

Of 121,505 patients awaiting GI endoscopy in January 2021, 45,529 (37.5%) were in the IPDC category, and 75,976 (62.5%) were in the planned procedure category. This total represents a 27.4% increase from January 2019, when the number of those awaiting an endoscopy was 95,386. An increase was seen every three months over the two-year period, with the largest single increase recorded from January 2020 to April 2020, a period coinciding with the first national ‘lockdown’, when 7,223 patients were added to the list.

**Figure 13.**
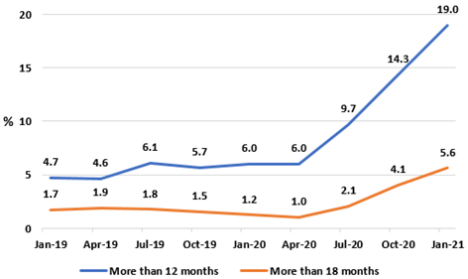
Percentages of IPDC GI endoscopy list waiting longer than 12 and 18 months (from National Treatment Purchase Fund).

As well as increasing in absolute terms, the proportion of patients in the ‘active’ IPDC waiting list category waiting long periods for an endoscopy rose in the two-year period analysed ( Figure 13), in particular after the onset of the pandemic. Having been relatively stable in 2019, the proportion of those on the IPDC endoscopy waiting list waiting more than 12 months rose from 6.0% in January 2020 to 19.0% in January 2021, and the proportion waiting more than 18 months from 1.2% to 5.6% in the same period.

Notably, at time of writing, length-of-wait data for endoscopy were only available for patients in the IPDC category and classified as ‘Active’, and not for patients in the planned procedure category. As such, the length of wait for around 70% of patients awaiting a scope nationwide remains uncharacterised. This constitutes a limitation of the data in this section.

## Discussion

This research set out to document trends in publicly available data on healthcare activity during the first nine months of the COVID-19 pandemic. Analysis was presented on three key areas of the Irish health system – primary care, community-based healthcare and the hospital. In this section, we discuss the potential implications of these findings for the implementation of Sláintecare.

### Primary care

The findings indicate that GPs played an instrumental role in Ireland’s COVID-19 response given that a majority (two-thirds) of all COVID-19 PCR tests were either referred by GPs, followed up on by GPs, or both. Moreover, the figures show that large numbers of telephone-based consultations were held with patients presenting with COVID-19 symptoms, with three peaks in this activity, each preceding the successive ‘waves’ of COVID-19. However, the number of patients availing of out-of-hours GP care fell significantly in 2020 coinciding with the first ‘lockdown’. This reflects other Irish and international literature that has highlighted changes in care-seeking activity during the pandemic, such as in emergency departments ( Brick *et al.*, 2020; Lazzerini *et al.*, 2020; Marron *et al.*, 2021).

Ireland’s primary care system has been described by international observers as inequitable ( Organisation for Economic Co-Operation and Development, 2019; Thomson *et al.*, 2014). Yet these trends represent significant changes in routes of access to healthcare, including in primary care, and speak to the extraordinary dedication and professionalism of healthcare workers. They also demonstrate the potential of the Irish healthcare system successfully to implement entirely novel approaches to healthcare at scale that are underpinned by universalism: the foundation stone of Sláintecare. Critically, all COVID-related healthcare in Ireland has remained free at the point of use ( Brick *et al.*, 2020); the public health benefit of minimising monetary barriers to healthcare access has been emphasised ( World Health Organisation, 2020b).

### Community-based healthcare

The analysis revealed that activity declined significantly in 2020 across multiple allied healthcare professions in the public system. Notably, the extent of the decline in activity varied between specialties. There may be several factors causing this variance, such as differences in adaptability to video-based or tele-consultations ( Tack *et al.*, 2021); workforce redeployment to tasks such as contact tracing ( Raidió Teilifís Éireann, 2020a; Raidió Teilifís Éireann, 2020b); and socioeconomic effects of the pandemic such as those related to childcare, particularly given that a majority of allied healthcare workers are women ( Del Boca *et al.*, 2020; Shannon *et al.*, 2019; U.S. Bureau of Labor Statistics, 2021).

Moreover, the proportion of infants receiving 10-month developmental screening checks on time from their PHN fell significantly in 2020. Further research documenting the long-term impact of the disruption to this element of early-childhood healthcare would be of value.

In addition to reduced activity, the findings point to significantly increased waiting list figures for the eight allied health specialties examined during the early months of the pandemic. Notably, these increases were immediately preceded by a significant reduction in total waiting list numbers in the first quarter of 2020 (see Figure 6). The reason for this decline is not clear. It may represent a true decline in numbers awaiting treatment, a statistical artefact arising from methodological change in list management, or a combination of both.

A core tenet of Sláintecare is the transition of healthcare from the acute hospital to the community. The reduced activity levels and increased waiting list figures documented here suggest that COVID-19 had a substantially negative impact on the levels of community-based healthcare delivered by the public healthcare system in Ireland in 2020. This lends considerable support to the assertion that the capacity for community-based healthcare should be bolstered and enhanced now and in the future; however, it also makes a strong case for ensuring that specialists are enabled, where possible, to remain in community settings if another wave of COVID-19 (or, indeed, a new health system crisis entirely) occurs in the future.

### Hospital care

The findings point to a significant disruption of scheduled hospital care during the COVID-19 pandemic, manifesting as increased waiting lists and longer wait times in the Irish healthcare system. The harms incurred to those experiencing exceptionally long waits for healthcare, in terms of delayed diagnosis and worsened health outcomes, have long been recognised as a policy concern globally ( Organisation for Economic Co-Operation and Development, 2019; Siciliani *et al.*, 2014; Wren, 2003). Indeed, reducing public hospital waiting list numbers in Ireland has been a stated priority of healthcare policy for decades and is a key imperative of Sláintecare ( Besley *et al.*, 2009; Burke *et al.*, 2019; Health Service Executive, 2021a; Health Service Executive, 2020a; Siciliani *et al.*, 2014).

However, this research indicates that the onset of COVID-19 has exacerbated already long hospital waiting lists for essential care in the Irish context, emphasising existing deficits in the current healthcare system that will need to be considered and addressed by policymakers. There are likely several factors at play here: most obviously, service suspensions leading to cancellations ( Government of Ireland, 2020b; National Public Health Emergency Team, 2020b), but also staff absences due to exhaustion and burn out ( Riley *et al.*, 2020), challenges to providing surgical care ( Bresadola *et al.*, 2020) and altered health-seeking behaviour patterns within the population, such as reduced rates of presentation to hospital related to fear of contracting the virus ( Brick *et al.*, 2020; Marron *et al.*, 2021).

If the implementation of Sláintecare is to be successful, measures to counteract or reduce the impact of these adverse effects in the longer-term, as well as protective mechanisms to prevent or mitigate similar consequences in the future, will need to be conceptualised, developed and put in place.

### Limitations and directions for future research

As mentioned at the outset, we performed a secondary analysis of publicly available data to examine trends in healthcare activity in Ireland during the first nine months of the COVID-19 pandemic. While secondary data analysis has clear value, this approach is not without limitations, the most notable of which is that researchers have to ‘make do’ with what they have (or what is available), rather than being able to gather tailored data.

Because of this, the findings presented here only represent activity in the public health system and not the private sector. Furthermore, as stated accordingly throughout, the findings likely provide an incomplete picture of healthcare activity as it relates to GPs, GI endoscopy waiting lists and community-based COVID-19 tests. Nevertheless, critical insights have been gleaned that warrant further research to improve our understanding of the impact of COVID-19 on Ireland’s health services and the potential implications for the implementation of Sláintecare.

Finally, certain consequences of the pandemic may take years to manifest fully. Moreover, the data utilised by this research were collected before several public health developments took effect, such as the introduction of walk-in COVID-19 testing centres and the roll-out of the national vaccination campaign. Further analyses of Irish healthcare activity should therefore endeavour to incorporate a longer observation period to enable stronger inferences to be made about the effect of the pandemic in the longer-term.

## Conclusion

The effect of the pandemic on the Irish health system has been profound. In hospitals and in the community, healthcare activity has been significantly disrupted. Moreover, the crisis is occurring in a context in which Ireland’s public health system has longstanding and complex issues regarding access to care and long wait-times that place us well behind the norm in other European and OECD countries ( Organisation for Economic Co-Operation and Development, 2019; Siciliani *et al.*, 2014; Wren, 2003). Many people have died of COVID-19, and the health toll of delayed and disrupted non-COVID care remains to be calculated. However, it is clear that reduced healthcare activity combined with increased waiting lists across the health system directly worsened access to healthcare during the pandemic. This contrasts with the Sláintecare aim of improved access to care.

Yet, Ireland’s health system responses to COVID-19 have also demonstrated a strong willingness to change, to adapt at speed and to implement novel solutions for healthcare delivery at scale. These advances carry the potential to improve healthcare access and provide a sense of what is achievable under Sláintecare’s vision for reform. To sustain this momentum will require continued emphasis on public health, the empowerment of individuals and communities, and support for those across the front-line of the health system. Prior to the pandemic, progress on Sláintecare implementation had slowed. However, the crisis has presented the Irish health system with a rare opportunity to harness the key lessons and progress of the pandemic and to ‘build back better’ toward a sustainable recovery post-COVID-19.

## Data availability

### Underlying data

This study undertook a secondary analysis of publicly available data, meaning that no primary data were collected as part of this research. Each data source is referenced throughout the paper with online websites and sources detailed. The main websites are also listed below:

   - https://www.sspcrs.ie/portal/annual-reporting
  This is an open-source data portal operated by the Primary Care Reimbursement Service (PCRS). Data obtained from this portal include figures on monthly eligibility levels for medical cards and GP visit cards. Figures on the number of COVID-19 telephone triages for which general practitioners submitted claims were also obtained from this portal.
   - https://www.hse.ie/eng/services/publications/performancereports/2020-performance-reports.html
  This provides access to the monthly performance reports published by the HSE in 2020. These reports contain data on community-based healthcare as well as out-of-hours GP care.
   - https://www.hse.ie/eng/services/publications/performancereports/2019-performance-reports.html
  This provides access to the monthly performance reports published by the HSE in 2019.
   - https://www.hse.ie/eng/services/publications/performancereports/2018-performance-reports.html
  This provides access to the monthly performance reports published by the HSE in 2018.
   - https://data.gov.ie/dataset/laboratorylocaltimeserieshistoricview1
  This resource is published by Ordnance Survey Ireland and contains data concerning national COVID-19 testing figures.
   -   National Treatment Purchase Fund: https://www.ntpf.ie/home/nwld.htm
         This database contains the national waiting list data.
