# Supplementary material for: Tracking aspects of healthcare activity during the first nine months of COVID-19 in Ireland: a secondary analysis of publicly available data
Source: HRB Open Res. 2024 Sep 13;4:98. Originally published 2021 Sep 2. [Version 3] doi: 10.12688/hrbopenres.13372.3 (PMC11439124; doi:10.12688/hrbopenres.13372.3)
Supplement: Peer review history

# Reviewer report: Reviewer response for version 3

**DOI:** 10.21956/hrbopenres.15327.r42408

**Published:** 2024-09-28

**Authors:** Sarah Rhodes

**Affiliations:** 1 The University of Manchester, Manchester, England, UK

The report appears much improved. I have no further comments.

Is the work clearly and accurately presented and does it cite the current literature?

Yes

If applicable, is the statistical analysis and its interpretation appropriate?

Yes

Are all the source data underlying the results available to ensure full reproducibility?

Yes

Is the study design appropriate and is the work technically sound?

Yes

Are the conclusions drawn adequately supported by the results?

Partly

Are sufficient details of methods and analysis provided to allow replication by others?

No

Reviewer Expertise:

I am a Medical Statistician. I work in trials, systematic reviews and epidemiological analysis of cohort data (relating to occupational risks of COVID-19).

I confirm that I have read this submission and believe that I have an appropriate level of expertise to confirm that it is of an acceptable scientific standard.

# Reviewer report: Reviewer response for version 3

**DOI:** 10.21956/hrbopenres.15327.r42409

**Published:** 2024-09-18

**Authors:** Michael Edmund O'Callaghan

**Affiliations:** 1 School of Medicine, University of Limerick, Limerick, Ireland

Well done to the authors on this latest version of this article. I'm happy to approve this version.

 I believe it makes a valuable contribution to the literature on understanding more about what happened "on the ground" in Ireland during the earlier years of the pandemic.

Is the work clearly and accurately presented and does it cite the current literature?

Yes

If applicable, is the statistical analysis and its interpretation appropriate?

Partly

Are all the source data underlying the results available to ensure full reproducibility?

Partly

Is the study design appropriate and is the work technically sound?

Yes

Are the conclusions drawn adequately supported by the results?

Partly

Are sufficient details of methods and analysis provided to allow replication by others?

Partly

Reviewer Expertise:

general practice, eHealth, open data, healthcare utilisation

I confirm that I have read this submission and believe that I have an appropriate level of expertise to confirm that it is of an acceptable scientific standard.

# Reviewer report: Reviewer response for version 2

**DOI:** 10.21956/hrbopenres.15013.r36403

**Published:** 2023-10-16

**Authors:** Sarah Rhodes

**Affiliations:** 1 The University of Manchester, Manchester, England, UK

(1) The sentence 'The complexity of these numerous subcategories creates difficulty both in capturing the full burden of patient wait times and in enabling comparisons with other jurisdictions' does not seem to belong in the 'data source' section. If there are complexities, please explain clearly what they are and how they were dealt with. In particular, could data for the same patient be repeated multiple times and with different sub-categorisation? Are there patients for whom the subcategory is unknown. The analysis section suggests that data were 'cleaned' - what exactly does this mean?

 (2) The analysis section is very brief. Please state which descriptive statistics were used, any graphs produced, how data were grouped/categorised (e.g. by month).

 (3) Please say more about the time periods used and why these vary from analysis to analysis.

 (4) Figure 3 includes 'covid cases nationally' - I don't see this data listed under data sources - please add. Please explain why you have chosen to add this to this graph.

 (5) Figure 5 is presented using 'cumulative patient numbers' - it would be helpful to know why. A more consistent approach to drawing graphs would help the reader as it takes a while to work out what is going on - monthly numbers as per previous figure may allow readers to see more granular detail.

 (6) Do figure 9 and table 4 show the same information? Maybe move one to an appendix.

 (7) As the word 'significant' has a particular meaning in statistics it might be best to remove 'significant' and 'significantly' from the discussion.

 (8) I'd recommend a conclusion that is more focussed on the findings from this piece of research rather than more general reflections/opinions. Rather than the sentence 'Yet, Ireland’s health system responses also revealed strong willingness and ability to adapt and to implement novel solutions for healthcare delivery, rapidly and at scale 'perhaps something like 'Irelands healthcare system saw the introduction/scaling up of telephone triage and COVID-19 testing by GPs suggesting a willingness to adapt'.

Is the work clearly and accurately presented and does it cite the current literature?

Yes

If applicable, is the statistical analysis and its interpretation appropriate?

Yes

Are all the source data underlying the results available to ensure full reproducibility?

Yes

Is the study design appropriate and is the work technically sound?

Yes

Are the conclusions drawn adequately supported by the results?

Partly

Are sufficient details of methods and analysis provided to allow replication by others?

No

Reviewer Expertise:

I am a Medical Statistician. I work in trials, systematic reviews and epidemiological analysis of cohort data (relating to occupational risks of COVID-19).

I confirm that I have read this submission and believe that I have an appropriate level of expertise to confirm that it is of an acceptable scientific standard, however I have significant reservations, as outlined above.

# Reviewer report: Reviewer response for version 2

**DOI:** 10.21956/hrbopenres.15013.r33694

**Published:** 2023-05-15

**Authors:** Michael Edmund O'Callaghan

**Affiliations:** 1 School of Medicine, University of Limerick, Limerick, Ireland

Thank you to the authors for their consideration of my feedback. I have considered their response and better understand the limitations of the datasets they were working with. I believe the article, particularly with the modified discussion and substantial limitations section, now better reflects the authors' findings.

 Just one issue I'd ask the authors to consider- in the Discussion paragraph:

 " *It appears that the health system sustained a ‘double hit’. Waiting lists grew during 2020, due to curtailed activity in hospitals. However, referral rates in real terms probably dropped, because of reduced availability of preventative care from GPs. This points to significant unmet need at community level. This has important policy implications.*"

 This is a very important point. However, I think the authors have the data in Table 4/Figure 9 to say with more certainty that referral rates dropped i.e. that numbers of patients in the 0-3 month category falling considerably (from 156k in Jan 2020 to 119k in Jul 2020) in the early stages of the pandemic was most likely due to the lack of new referrals entering the system. i.e. it is unlikely that the numbers dropped considerably as patients in the 0-3mth category were suddenly prioritised over those waiting years etc. This sticks out as a trend not seen elsewhere in these data. Patients clearly are leaving this 0-3mth category to enter the 3-6mth category (with a small number perhaps being seen/removed from the waiting list over the period in question), but are not being replaced as per usual.

 The reasons as to why are most likely due to "reduced availability" of GP services, although mention of reduced healthcare seeking behaviour from patients is also likely to be more reflective of the complete picture.

 Finally, the ordnance survey reference I mentioned in my previous report now doesn't have a Reference Source link. Perhaps this was the authors' own analyses based on data provided by OSI? Or the CSO perhaps? If so this should be clearly stated in the References section. As the source material for Figure 4 this should be rectified.

 In conclusion, I couldn't agree more with the authors' assertions that better data collection and use of said data is critically important for improving research and service delivery (for pandemic related issues and for healthcare in general), I hope this study contributes to the growing calls for this.

 Kind regards,

 Dr Mike O'Callaghan 3/5/23

Is the work clearly and accurately presented and does it cite the current literature?

Yes

If applicable, is the statistical analysis and its interpretation appropriate?

Partly

Are all the source data underlying the results available to ensure full reproducibility?

Partly

Is the study design appropriate and is the work technically sound?

Yes

Are the conclusions drawn adequately supported by the results?

Partly

Are sufficient details of methods and analysis provided to allow replication by others?

Partly

Reviewer Expertise:

general practice, eHealth, open data, healthcare utilisation

I confirm that I have read this submission and believe that I have an appropriate level of expertise to confirm that it is of an acceptable scientific standard, however I have significant reservations, as outlined above.

# Reviewer report: Reviewer response for version 1

**DOI:** 10.21956/hrbopenres.14564.r32093

**Published:** 2022-05-23

**Authors:** Michael Edmund O'Callaghan

**Affiliations:** 1 School of Medicine, University of Limerick, Limerick, Ireland

This is an interesting and novel study that uses open data to describe some aspects of the Irish national response to COVID-19, from HSE-held data derived from general practice, allied healthcare in the community, and national treatment purchase fund reports.

 While the authors have had to go to considerable lengths, particularly for the NTPF data, to contextualise and explain what exactly it is they are presenting, this article, in my view, requires a major revision.

 My primary issue is that the data concerned with general practice mainly concentrate on COVID-19 related work, while the other areas (allied healthcare in the community and waiting list data) pertain to non-COVID work.

**GP DATA**

 For these GP-related data, out-of-hours data is very interesting. Also, the finding that there is a flurry of activity Re: COVID telephone consultations before COVID-19 peaks is interesting (if what one might expect!).

 However, it is not clear where the data relating to referrals for COVID-19 testing is originating from. I’m afraid the reference link for Ordnance Survey Ireland is not working as of 22-5-22. Perhaps this is now data held by the CSO data hub? In any case, it is difficult to say how many referrals for testing came from general practice, as public health and GP referrals seem to both be recorded as “community” referrals (as noted by the authors in Page 6 – “may over-state GPs’ role in referrals for testing”).

 How much this issue affects the overall conclusions is uncertain. However, from data presented in the article GPs made claims for 413k COVID calls in Dec 20 compared to 174k in Jan 21 (Figure 3). For the same 2 months, from Figure 4, GPs seem to be responsible for 300k tests in Dec 20 and ~ 500k tests in Jan 21. This does not make sense entirely as if GPs had sent 500k people for tests in Jan 21 they would have claimed in excess of this number. While PCRS may not always receive or process GP claims rapidly, eReferrals for testing presumably can be handled more quickly than old paper-style claims. In any event, I don’t see a large number of old claims being processed in early 2021 that might explain this discrepancy. Of note out-of-hours GP services have been reimbursed very differently during the pandemic (e.g. no longer being paid per patient, thereby, unfortunately, making comparisons with previous years via PCRS reports for OOH claims impossible), so referrals for testing from these important services might not feature in Figure 3.

 As the authors note, GPs have been able to make claims for telephone consultations relating to COVID-19 symptoms (using the code “CB”) since the early stages of the pandemic. We have also been able to claim for in-house respiratory consultations (using the code “CA”). Access to these data, if possible, could allow more definitive description of both of these aspects of the general practice’s response to the pandemic.

 Furthermore, perhaps PCRS data, which pertains to both COVID and non-COVID work could be used to explore how much regular daytime general practice continued while allied healthcare in the community and NTPF data suggest other aspects of the public healthcare system in Ireland faltered.

 If there is a large difference in the volume of work general practice, in keeping with that seen in the other settings mentioned, then that is an important finding.

 However, if it is shown that general practice experienced less of a dip than other areas, then that, in my view is an even more important finding i.e. why was general practice different? This might allow the authors to build their discussion around Sláintecare proposals that seek to harness what it is that works in primary care in Ireland.

 While I appreciate it is difficult to access data from general practice EMRs (and that both GPs and academic depts/colleges/researchers need to do more to encourage this), there are other data sources publicly available that may be of interest e.g. vaccine rates for the under 2s from DOH key trends [Table 4.9 https://www.gov.ie/en/publication/350b7-health-in-ireland-key-trends-2021/], PCRS payments to pharmacists (largely from GMS prescriptions which are only issued by GMS GPs), in addition to important datasets held by the HSE including chronic disease management data, COVID-19 vaccine data, that may illustrate some of the overall activity in general practice settings in RoI in the past 2 years.

**ALLIED HEALTH DATA**

 I believe this is the strongest section presented. Allied health data in the community sections are presented well and unfortunately for the Irish Health service, are a clear description of yet another inadequate, unfair, and worsening waiting list situation the Irish public without healthcare insurance or the means to pay privately must endure. It is noted from Table 2 in this Section that many allied healthcare professional waiting lists experienced a sharp decrease in early 2020, again perhaps to “waiting list validation” exercises.

**NTPF**

 NTPF data presented from page 10 to 13 is similarly troubling in terms of overall trends, e.g. Table 6.

 However, unfortunately, it is difficult to appraise what is happening to patients who have been referred, and when it happens, from NTPF data presented.

 I don’t believe this is the author's fault, it merely represents some of the inadequacies of the data sources examined. Table 4 deserves particular mention here, where NTPF data presented are in need of clarification, presumably by the NTPF themselves.

 Specifically, the jump seen in the number in the “15-18 months” category between Oct-19 and Jan-20 is unusual. If bands are contiguous, then the overall number of “new people” spilling into a 3 month waiting list category (15-18mths) over a 3 month gap surely cannot exceed that in the preceding waiting list category (12-15mths), unless a huge number of patients are “found” by being reclassified elsewhere? At several points in this article, including for the Allied healthcare data, big changes happen at the end of 2019 in many waiting lists, which requires more in terms of discussion, in my opinion.

 In fairness to the authors, they allude to potential “statistical artefact” in Page 16 Paragraph 2. However, the issues outlined here, coupled with the lack of data on procedures being carried out on the NTPF, the lack of disclosure on “waiting list validation” exercises, etc., and other matters such as the use of the cross-border directive, etc., lead me to believe NTPF data output is not researcher-friendly in its current form.

 While the authors note the significant limitations and complexities of NTPF data (page 4, second last paragraph), I wonder if an approach to concentrate on a smaller subset of NTPF data +/- inclusion of more acute hospital data might allow more definitive statements to be made on the effect of COVID-19 on public secondary healthcare in Ireland.

 With that said, I do find it interesting that numbers in the 0-3mth category in Table 4 decreased considerably in mid 2020. This would be consistent with people not being referred in by general practice, as noted by the authors (Page 3, 4 ^th^ paragraph). While I have stated above my reservations about making firm conclusions about the other data presented in Table 4 (i.e. the longer wait times), it is abundantly clear that the number of waiting lists still grew considerably overall during 2020. This is something of a double-hit issue, where there is a large unmet need in the community (as they’re not coming in to be referred) and in those currently on the waiting list for older issues that are not being dealt with by the public system or the NTPF, which does not bode well for the waiting list numbers in the near future.

**DISCUSSION**

 Points on GP OOH and "Universalism" are well made.

 The point on keeping allied healthcare professionals in their posts in the future is interesting, though it would be stronger if we knew how many were redeployed. Highlighting the need for bolstering the existing workforce in allied healthcare professionals could be made stronger by referencing the pre-pandemic situation described by Table 2- (i.e. many specialties with a quarter or more patients on the waiting list being on it > 1yr).

 Discussion on hospitals could allude to the unmet need mentioned in the paragraph above ("double-hit issue"), though perhaps the authors would need to gather more evidence before this could be described with more conviction.

**REVIEWER CONCLUSION**

 This is a well written article that is considerable in its scope. However, I feel it needs some work to more clearly define data sources, which will strengthen the discussions/conclusions considerably.

 I hope the authors will agree with at least some of the points I have made and I hope they will be agreeable to redrafting the article +/- a response in the near future.

 Work to highlight how far we need to go as a country with respect to publicly available sources of healthcare data is critical in my view.

 Many thanks,

 Dr. Mike O Callaghan

 mike.ocallaghan@icgp.ie

Is the work clearly and accurately presented and does it cite the current literature?

Yes

If applicable, is the statistical analysis and its interpretation appropriate?

Partly

Are all the source data underlying the results available to ensure full reproducibility?

Partly

Is the study design appropriate and is the work technically sound?

Yes

Are the conclusions drawn adequately supported by the results?

Partly

Are sufficient details of methods and analysis provided to allow replication by others?

Partly

Reviewer Expertise:

general practice, eHealth, open data, healthcare utilisation

I confirm that I have read this submission and believe that I have an appropriate level of expertise to state that I do not consider it to be of an acceptable scientific standard, for reasons outlined above.
